# Decentralised Oncology Trials in the UK: Evidence, Regulation and Academic Clinical Trials Units’ Perspectives

**DOI:** 10.1136/bmjonc-2026-001145

**Published:** 2026-08-20

**Authors:** Oyeyemi Akala, Harriet S Walter, Claire Snowdon, Gianina-Ioana Postavaru, Judith M Bliss, Hannah Gribble, Abigail Botschka, Fay H Cafferty

**Affiliations:** 1 Clinical Trials & Statistics Unit (ICR-CTSU), The Institute of Cancer Research, London, UK; 2 Leicester Cancer Research Centre, University of Leicester, Leicester, UK; 3 CRUK/NIHR Leicester Experimental Cancer Medicine Centre, University of Leicester, Leicester, UK; 4 School of Psychology and Vision Sciences, University of Leicester, Leicester, UK

**Keywords:** Clinical Trial, Neoplasms, decentralised clinical trial, clinical trial methodology, health policy

## Abstract

**Objectives:**

Decentralised clinical trials (DCTs) are increasingly promoted in UK research policy and practice; however, their benefits and implementation challenges in oncology remain unclear. We examined DCT use in oncology through a review of studies evaluating decentralised oncology trial delivery, UK regulatory and policy frameworks and a survey of UK academic Clinical Trials Units (CTUs).

**Design:**

Multimethod study including a structured literature review, policy review and cross-sectional survey of UK Clinical Research Collaboration (UKCRC)-registered CTUs.

**Setting:**

International literature, UK-based regulatory and policy documents and survey data from UKCRC-registered CTUs.

**Interventions:**

None.

**Main outcome measures:**

Literature review: decentralised components evaluated and reported outcomes.

Policy review: opportunities, barriers and gaps in UK regulatory and policy guidance relevant to DCTs.

Survey: CTU awareness, uptake, perceived benefits and challenges related to decentralised trial delivery, comparing oncology and non-oncology settings.

**Results:**

Of 68 studies meeting broader eligibility criteria, only 8 formally evaluated decentralised elements within oncology trials and were included in the primary synthesis. Conducted across five countries, these studies primarily assessed feasibility, recruitment, usability and data completeness. Only one UK oncology study formally evaluated DCT methods, assessing use of remote electronic consent to support recruitment during the COVID-19 pandemic, highlighting limited published UK evaluation. Non-UK studies showed mixed findings, including improvements in trial processes or participant satisfaction in some contexts alongside operational challenges in others.

The UK-based policy review identified strategic support for decentralisation but fragmented regulatory guidance and ambiguity around key decentralisation concepts. Temporary COVID-19-induced flexibilities demonstrated feasibility but have not been fully incorporated into permanent guidance.

Survey responses were received from 27 CTUs (54% response rate). Awareness of decentralised approaches was high; however, use of these was lower in oncology than in non-oncology trials. Decentralisation was most commonly applied to patient follow-up. CTUs reported reduced participant burden and increased flexibility, but also challenges relating to governance, contracting, infrastructure, staffing and data management.

**Conclusions:**

Decentralised approaches in UK oncology trials are gaining momentum but remain unevenly adopted, alongside a limited oncology-specific evidence base, regulatory ambiguity and operational challenges. Oncology presents distinct challenges that may limit decentralisation of trials compared with other disease areas, including complex interventions, intensive safety monitoring requirements and protocol-specific assessments. Evidence on patient/public involvement and acceptability of decentralised approaches among people with cancer remains limited. Clearer guidance and more robust evaluation, incorporating patient perspectives, are needed to support wider implementation in the UK and may also be relevant internationally.

**WHAT IS ALREADY KNOWN ON THIS TOPIC**

- Many clinical trials already conduct selected activities away from conventional in-person patient attendance at investigational sites, and awareness and interest in more decentralised approaches increased substantially during the COVID-19 pandemic. Evidence evaluating how these approaches have been implemented and assessed, particularly within UK oncology trials, remains limited.

**WHAT THIS STUDY ADDS**

- This study examined the evidence base for use of decentralised approaches, as well as their regulation and implementation in UK oncology trials. Despite widespread awareness, adoption remains uneven and lower than in non-oncology settings, with use most commonly in patient follow-up. Fragmented regulatory guidance and broader operational challenges reported by CTUs relating to governance, contracting, infrastructure, data management and staffing were identified as key factors that may limit wider implementation.

**HOW THIS STUDY MIGHT AFFECT RESEARCH, PRACTICE OR POLICY**

- These findings highlight a need for clearer, consolidated regulatory guidance and further evaluation of decentralised methods in oncology. Greater incorporation of cancer patient perspectives may also help inform acceptable and practical approaches to implementation in the UK and more broadly across healthcare systems.

## Introduction

 Cancer clinical trials are typically organised around hospital-based (site) models of delivery. While this approach supports established operational processes, it may prioritise traditional ways of working over patient accessibility. Requirements for in-person participant visits can create barriers for individuals facing geographical, logistical or health-related issues, potentially limiting recruitment and affecting the representativeness and generalisability of trial findings.^1^ Although some aspects of trial participation already occur outside investigator sites, many core trial processes continue to rely on in-person participant attendance. Addressing these barriers is recognised as a key step in improving access to trials and ensuring that study populations better reflect the patients seen in routine practice.

A 2023 government-commissioned review of UK commercial clinical trials, led by Lord O’Shaughnessy, highlighted declining UK trial activity and structural barriers including the continued reliance on hospital-based research models, a challenge relevant to both academic and commercial research.^2^ UK government strategy, outlined in *Saving and Improving Lives: The Future of UK Clinical Research Delivery*, emphasises integrating research into routine care and leveraging innovative technologies to recruit and support patients ‘where they are’, with the aim of improving access and reducing reliance on hospital-based visits.^3^ This policy direction aligns with broader reforms to UK research infrastructure and National Health Service (NHS) strategy aimed at expanding healthcare delivery beyond hospital sites and into community-based settings.^4^

Decentralised clinical trials (DCTs), in which activities traditionally conducted at investigator sites are delivered through remote, local or hybrid models, have emerged as a potential response to these challenges. Supported by digital tools and alternative models of delivery, DCTs aim to centre trial activities around participants and may reduce travel burden and improve accessibility.^5^ DCTs are variably defined in the literature, with terminology including remote, virtual, teletrials, digital, patient-centric and flexible trials often used interchangeably, reflecting ongoing inconsistency in how these approaches are described.^5^ In this study, we apply a broad definition of decentralisation, recognising that it exists along a spectrum from individual remote components to fully remote trial designs.

Despite increasing adoption of decentralised approaches and publication of regulatory guidance on decentralised elements in clinical trials,^6,7^ oncology-specific evidence evaluating their impact remains limited. The Trials@Home consortium, for example, concluded that the available evidence was insufficient to determine whether DCTs improve recruitment, retention or cost efficiency, with only one oncology trial identified among the trials included in their quantitative analysis.^8^ Importantly, the 2022 review was not oncology-specific, did not examine practical implementation experiences and preceded recent regulatory and policy developments in this rapidly evolving area.

The COVID-19 pandemic disrupted cancer trial delivery globally, prompting rapid streamlining of procedures, adoption of remote assessments and patient-centred approaches.^9^ These adaptations demonstrated the feasibility of delivering some trial activities outside traditional site-based models and have stimulated interest in more flexible and decentralised methods. However, within the UK, there remains limited published evidence on feasibility and implementation of decentralised approaches in oncology, particularly in relation to regulatory considerations and their interpretation in practice. This study addresses this gap by examining: (1) the current international evidence base evaluating the implementation and impact of decentralised approaches in oncology trial delivery, (2) relevant UK policy and regulatory guidance and (3) the perspectives of UK academic Clinical Trials Units (CTUs) on decentralised oncology trials.

## Methods

This study comprised three complementary strands to examine decentralised oncology trials within the UK context. First, a structured international narrative literature review using systematic search and screening methods was undertaken to identify oncology trials that have evaluated decentralised approaches (‘what is known’). Second, a targeted review of legislation and policy documents was conducted to map the current regulatory framework and guidance relevant to DCT methods (‘what is allowed’). Third, a survey of UKCRC-registered CTUs was administered to capture current adoption of, and experiences with, decentralised approaches (‘what is happening in practice’).

### Literature review (what is known)

Studies were eligible if they involved adults ≥18 years with solid tumours or populations at risk of developing solid tumours and explicitly evaluated at least one decentralised or digitally enabled component of clinical trial delivery (e.g., recruitment, consent, data collection, monitoring or follow-up delivered remotely or outside traditional investigator sites). For the purposes of this review, ‘evaluation’ referred to formal assessment of the impact of decentralised approaches on trial-related outcomes, for example, recruitment, retention, data completeness, feasibility or participant experience. Both interventional and observational studies (embedded within trials) were eligible where they included prospective or retrospective evaluation of decentralised trial delivery.

Studies were excluded if they focused on paediatric populations, haematological malignancies, examined behavioural, psychosocial, educational or exercise interventions (as the review focused on screening, diagnostic, treatment, procedural and device trial settings), were not published in English, did not explicitly evaluate the decentralised component, or were systematic reviews.

An initial search was piloted in July 2024. Search terms (online supplemental file 1) were refined using the Trials@Home consortium systematic review^8^ as a reference. Comprehensive searches were conducted from database inception to October 2025 across MEDLINE, EMBASE, EMCARE and HMIC. Grey literature searches were undertaken using OpenGrey and Web of Science and forward and backward citation searching was conducted in Google Scholar. The ClinicalTrials.gov registry was also searched using combinations of ‘oncology’ with terms such as ‘virtual’, ‘remote’, ‘decentralised’ and ‘decentralized’.

Titles and abstracts were screened against the eligibility criteria, with full texts retrieved for potentially relevant studies. For consistency of screening decisions, two additional reviewers independently screened random samples of titles and abstracts (approximately 10% each), with discrepancies resolved through discussion. Data extraction was conducted by the first author using a structured Excel spreadsheet. Extracted fields included bibliographic details, study characteristics, decentralised components, evaluation outcomes (eg, recruitment, retention, data completeness), as well as other reported facilitators, benefits and challenges (online supplemental file 2). Findings were summarised descriptively and synthesised narratively. We describe this as a structured literature review rather than a systematic review because the aim was to map evidence in an emerging, heterogeneous area. Systematic search and screening methods were used to improve transparency and reproducibility, but the review was not prospectively registered. Formal quality or risk-of-bias assessment was not undertaken because many studies reported feasibility, implementation or trial-process outcomes without formal comparators or statistical analyses, limiting the applicability of existing risk-of-bias tools. The synthesis was descriptive and was not designed to generate pooled estimates or make comparative effectiveness claims; key methodological limitations are therefore described narratively.

### Legislation and policy review (what is allowed)

A review of legislation, regulatory guidance and policy documents relevant to decentralised trial delivery in the UK was undertaken. The end-to-end clinical trial delivery process was mapped and categorised into domains with potential for decentralisation or requiring specific regulatory consideration: trial set-up, recruitment, intervention delivery and participant follow-up, data collection and handling and operations (including oversight and monitoring). Within each domain, specific activities of interest were identified, such as site initiation visits, consent, investigational medicinal product (IMP) administration, among others.

UK regulatory authority websites (including the Medicines and Healthcare products Regulatory Agency (MHRA), Health Research Authority (HRA) and Department of Health and Social Care) were then searched for relevant legislation, regulations and guidance, alongside policy papers addressing decentralised trials. A snowballing approach was used to identify additional relevant documents cited within included sources. Searches and screening were conducted between November and December 2024, with an updated search in December 2025. No further relevant sources were identified.

A structured Microsoft Word form (online supplemental file 3) helped to extract the data, including issuing authority, relevant provisions and associated opportunities and barriers for decentralised delivery. Extracted data were synthesised narratively across domains to identify areas of alignment, uncertainty and gaps.

### Survey of UKCRC CTUs (what is happening in practice)

A 46-item survey (online supplemental file 4) was developed iteratively, combining closed-ended questions with open-ended free-text items. The survey introduction included a working definition of DCTs as a spectrum ranging from individual remote trial components to fully decentralised models, with examples including remote consent, use of telemedicine and wearable technologies. Item development was informed by relevant literature, UK policy and regulatory guidance on decentralised trials and practical considerations identified through the study team’s experience of designing and delivering oncology clinical trials.

The survey aimed to evaluate how decentralised approaches have been integrated within trials conducted by UK academic CTUs, identify levels of awareness, perceived benefits and challenges and compare uptake and experiences between oncology and non-oncology settings. No formal psychometric validation or cognitive interviewing was undertaken, as this was a one-off survey designed to collect descriptive information from CTU staff. However, content validity was supported through iterative review by the multidisciplinary study team, which included a cancer CTU Operations Director, a Principal Statistician and a Consultant Medical Oncologist. Face validity was assessed through pilot testing with two senior operational colleagues within the study team’s CTU, with feedback informing revisions to question wording and order, terminology, explanatory text, oncology/non-oncology distinctions and free-text instructions.

The target population comprised the 49 CTUs registered with the UKCRC at the time of the survey. The survey was distributed electronically via the UKCRC Registered CTU Network secretariat, with target respondents being Operations Directors and Quality Assurance Managers. CTUs with both oncology and non-oncology teams that function separately were permitted to submit two responses, which were analysed separately.

The survey was delivered through Jisc online surveys^10^ and remained open for 12 weeks from 7 October to 23 December 2024. The invitation email described the purpose of the survey, estimated completion time and the option to provide an email address if respondents wished to be acknowledged in any resulting publications. As the survey targeted senior CTU staff in their professional roles and did not collect sensitive personal data, it was conducted as a service evaluation/stakeholder consultation and formal ethical approval was not required. Participation was voluntary, and completion of the survey was taken to indicate implied consent.

Quantitative responses were summarised descriptively using frequencies and percentages to characterise awareness, adoption and use of decentralised trial elements across CTUs. Free-text responses were analysed thematically using an inductive approach, with coding conducted by the first author using NVivo (v15, QSR International) and cross-checked with author GP through review of the coding framework, emerging themes and a sample of responses. Themes were broadly organised around reported experiences of decentralised trial delivery, including considerations, perceived benefits, challenges and adaptations, rather than being formally organised by trial processes.

## Results

### Literature review

**Figure 1:**
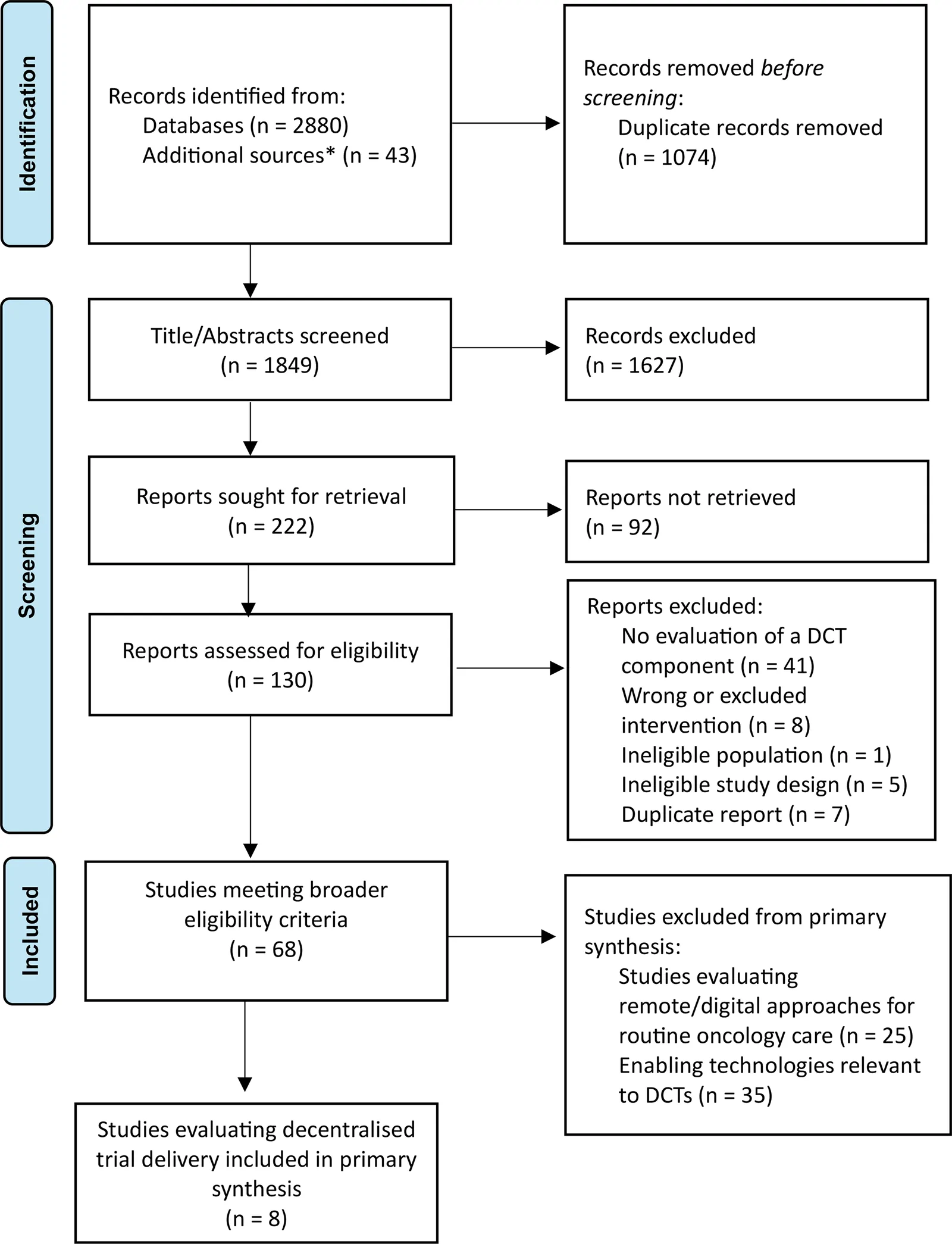
PRISMA flow diagram of article selection. *Additional sources included searches of Web of Science and OpenGrey. DCTs, decentralised clinical trials.

The search identified 2923 records (2880 from databases and 43 from other sources; Figure 1). After removal of duplicates, 1849 records remained for title and abstract screening, of which 222 reports were sought for retrieval. Reviewer agreement at title and abstract screening was high. A second reviewer screened 260 records, of which 6 were classified as ‘maybe’ and excluded from analysis; agreement with OA was 250/254 (98.4%) among records with definitive decisions. A separate sample of 282 records was screened by a third reviewer; after excluding 14 ‘maybe’ decisions and 8 duplicates, agreement with OA was 240/260 (92.3%). Discrepancies mainly involved borderline studies evaluating enabling technologies, digital oncology tools or remote care approaches evaluated for routine oncology care rather than decentralised trial delivery and were resolved through discussion and consensus.

A total of 130 reports were successfully obtained and assessed for eligibility while 92 reports could not be retrieved, most commonly because only conference abstracts were available and no additional study information could be identified elsewhere. Where full-text publications were unavailable, supplementary information was sought from trial registries and/or study websites.

Of the 130 reports assessed for eligibility, 68 were considered relevant to decentralised approaches (figure 1). However, only eight studies explicitly evaluated decentralised elements within oncology clinical trials and formed the primary focus of the synthesis (table 1). Studies were conducted in the USA (n=4), UK (n=1), Japan (n=1), Sweden (n=1) and the Netherlands (n=1), and included a mix of academic and industry-sponsored trials. Study size ranged from early feasibility studies enrolling fewer than 10 participants to large pragmatic trials planning to recruit up to 100 000 participants, with study periods spanning 2014–2025. Outcomes assessed included feasibility, recruitment, access, acceptability, usability and data completeness.

**Table 1:** Oncology studies evaluating decentralised methods to improve trials methodology

First author (year)	Study/acronym (registry ID)	Population (country)	Intervention	Decentralised elements	DCT comparator	Outcomes evaluated	Main findings	Status
Valachis *et al*^19^ (2024)	TELEPIK (NCT04862143)	HR+/HER2- PIK3CAm advanced breast cancer (Sweden)	Alpelisib+Fulvestrant	Remote AE monitoring via mobile platform; telemedicine	None	Feasibility (accrual, acceptability)	Early termination due to poor accrual; reported barriers included lack of IT integration with hospital infrastructure and unclear division of responsibilities between investigator and treating physicians	Terminated early
Brown *et al*^12^ (2024)	SHORT-FOX (NCT04380337)	Rectal cancer (USA)	Short-course RT+FOLFOXIRI	Local chemotherapy with telehealth AE review	Chemotherapy delivered at investigator site	Access, travel burden, equity indicators	Local chemotherapy reduced travel distance, time and costs. Participants opting for local treatment had higher ADI scores	Completed
Taniguchi *et al*^11^ (2023)	WJOG15221M/ ALLBREAK (jRCT2041210148)	Rare ALK+tumours (Japan)	Brigatinib	Remote eConsent, telemedicine, home drug delivery, local imaging	None	Feasibility, access	Six patients enrolled (four fully remote). No major protocol deviations reported; decentralised delivery enabled participation of patients who might otherwise not have accessed the trial	Not recruiting
Almeida-Magana *et al*^14^ (2022)	NeuroSAFE PROOF (NCT03317990)	Localised prostate cancer (UK)	NeuroSAFE RARP vs standard RARP	Remote eConsent; remote PROMs	Preimplementation study processes	Recruitment, data completeness	Recruitment and follow-up maintained during COVID-19; few missing primary outcome data	Active, not recruiting
Choy *et al*^13^ (2022)	WISDOM (NCT02620852)	Breast cancer screening trial (USA)	Risk-based vs annual screening	Online recruitment, consent, enrolment, saliva kits, etc	Preimplementation study processes	Enrolment conversion, data completeness	Streamlined online processes improved conversion from consent to enrolment and increased questionnaire completion and data availability	Recruiting
Paulissen *et al*^16^ (2022)	N/A	Patients with cancer in RT trials (Netherlands)	Digital previsit questionnaire	Remote previsit symptom reporting via mHealth app	None	Usability, visit efficiency	High usability (mean SUS 86.8). Patients and researchers reported improved visit preparation and efficiency; no data loss reported	Complete
Kurzrock *et al*^17^ (2021)	ALPHA-T (NCT04644315)	ALK+solid tumours (excl. NSCLC) (USA)	Alectinib	Remote consent, home nurse visits, IMP shipment, telemedicine	None	Feasibility, accrual	Planned enrolment of 50; only one patient enrolled. Trial terminated due to difficulty identifying and recruiting rare molecular subgroups	Terminated early
Galsky *et al*^15^ (2017)	M-RePoRT (NCT02376166)	Castration-naive prostate cancer (USA)	Metformin	Telemedicine visits; remote safety and QoL assessments; local labs	None	Feasibility, acceptability	100% completion of eligible telemedicine visits; high patient satisfaction and willingness to participate in future telemedicine trials	Completed

ADI, Area Deprivation Index; AEs, adverse events; ALK, anaplastic lymphoma kinase; eConsent, electronic consent; HER2−, human epidermal growth factor receptor 2 negative; HR+, Hormone receptor positive; IMP, investigational medicinal product; IT, information technology; NSCLC, non-small cell lung cancer; PIK3CAm, PIK3CA mutation; PROMs, patient-reported outcome measures; QoL, quality of life; RARP, robotic-assisted radical prostatectomy; RT, radiotherapy; SUS, system usability scale; Univ, University; WJOG, West Japan Oncology Group.

The remaining 60 studies were excluded from the main synthesis as they evaluated either remote or digital activities in routine oncology care (n=25) or enabling technologies relevant to decentralised trials (n=35). Examples included implementation of electronic symptom-monitoring programmes in routine oncology care and studies evaluating artificial intelligence-based methods for extracting cancer outcomes from electronic health records. While relevant to decentralised trials, these studies did not evaluate decentralised trial delivery itself. However, they may still provide useful contextual insights into the implementation and use of specific decentralised approaches in practice.

Across the eight methodology-focused studies, decentralised approaches were typically implemented alongside conventional site-based trial activities, with specific elements delivered remotely. The WJOG15221M/ALLBREAK study was described by its authors as Japan’s first fully decentralised oncology trial. This incorporated telemedicine consultations, remote consent, home delivery of the investigational drug and locally delivered blood tests and imaging.^11^ Although some procedures were conducted within partner hospitals, participants did not attend the central investigator site.

Approaches used to evaluate decentralised trial elements varied across studies. None of the studies randomised participants to decentralised versus conventional trial delivery. Some studies used before and after implementation comparisons, or single-group feasibility assessments without concurrent comparators. Only one study included a contemporaneous comparator. In the SHORT-FOX trial, travel burden and participant characteristics were compared between participants choosing to receive chemotherapy locally and those treated at the central study site. Local chemotherapy delivery combined with telehealth adverse-event review was associated with reduced travel distance, time and costs, and participants with higher Area Deprivation Index scores were more likely to opt for local treatment.^12^

A number of studies relied on before-and-after comparisons following the introduction of decentralised processes. For example, the WISDOM breast screening trial reported improved conversion from consent to enrolment, increasing from 78% to 93% and higher questionnaire completion (75% vs 59%) following implementation of streamlined online recruitment and enrolment processes.^13^ Similarly, the NeuroSAFE PROOF trial showed that remote electronic consent (eConsent) and electronic patient-reported outcome measures (ePROMs) collection enabled recruitment to continue during the COVID-19 pandemic, with 63 participants recruited following implementation and only three participants requesting paper consent forms.^14^

Other studies assessed decentralised trial components without formal comparator groups. The M-RePORT trial replaced onsite visits with telemedicine consultations in an interventional prostate cancer study, achieving completion of all 84 eligible telemedicine visits and high patient satisfaction.^15^ A radiotherapy study in the Netherlands also reported high usability of a mobile health application for symptom reporting, improving trial visit preparation and efficiency.^16^

In some cases, early study termination limited the ability to formally evaluate the impact of decentralised trial delivery. Two studies were discontinued due to low accrual. The ALPHA-T trial,^17^ which targeted an ultra-rare patient population, was discontinued primarily because of recruitment difficulties rather than decentralisation itself. Although the decentralised model aimed to expand access to geographically dispersed participants, it did not overcome the challenges of identifying and enrolling eligible participants with rare molecular alterations.^18^

The TELEPIK study highlighted operational challenges associated with implementing decentralised trial elements. New digital systems were not integrated into existing hospital infrastructure creating additional workload for research teams. The study also reported uncertainty regarding clinical responsibilities between local physicians and trial investigators.^19^

Overall, the included studies used heterogeneous approaches, primarily evaluating feasibility, implementation or trial process outcomes; formal statistical analyses were limited, meaning potential sources of bias and confounding were generally not addressed.

### Legislation and policy review

Twenty documents were identified (table 2), comprising five legislative texts,^20–24^ nine guidance documents,^25–33^ two COVID-19-specific guidance documents,^34,35^ three policy papers^2,3,36^ and one position statement.^37^

**Table 2:** Regulatory, guidance and policy documents relevant to decentralised clinical trials in the UK

Category	Document	Issuing organisation	Year
Primary legislation	The Medicines for Human Use (Clinical Trials) Regulations (SI 2004 No. 1031)	UK Government	2004
	Electronic Identification and Trust Services for Electronic Transactions (Amendment, etc) (EU Exit) Regulations 2019 (SI 2019 No. 89)	UK Government	2019
	Medical Devices Regulations 2002 (SI 2002 No. 618)	UK Government	2002
	Data Protection Act	UK Parliament	2018
	UK General Data Protection Regulation	UK Government	2018
Guidance relevant to decentralised elements of interest	ICH harmonised guideline integrated addendum to ICH E6(R1): Guideline for Good Clinical Practice ICH E6(R2)	ICH	2016
	Set up research activity at NHS organisations (interventional research)	HRA	2023
	Oversight and monitoring activities	MHRA	2022
	Joint statement on seeking consent by electronic methods	HRA/MHRA	2018
	Risk-Adapted Approach to clinical trials and Risk Assessments	MHRA	2022
	Good manufacturing practice and good distribution practice	MHRA/DHSC	2014
	Regulating medical devices in the UK	MHRA	2020
	Position Statement and Guidance: Electronic Health Records	MHRA	2015
	Access to Electronic Health Records by Sponsor representatives in clinical trials	MHRA	2020
Guidance specific to the COVID-19 pandemic	Managing clinical trials during COVID-19	MHRA	2020
	Making changes to a research study to manage the impact of COVID-19	HRA	2021
Policy	Commercial Clinical Trials in the UK: the Lord O’Shaughnessy review	DHSC	2023
	Saving and Improving Lives: The Future of UK Clinical Research Delivery	DHSC	2021
	The Future of Clinical Research Delivery: 2022 to 2025 implementation plan	DHSC	2022
Position statement	Decentralised trial methods position statement	HRA	2023

DHSC, Department of Health and Social Care; HRA, Health Research Authority; ICH, International Council for Harmonisation; MHRA, Medicines and Healthcare products Regulatory Agency.

Core legislation included the *Medicines for Human Use (Clinical Trials) Regulations 2004*,^20^ the *Electronic Identification and Trust Services Regulations 2019*, the *Medical Devices Regulation 2002*,^21^ the *Data Protection Act 2018*^23^ and the *UK General Data Protection Regulation 2018*.^24^ Guidance documents addressed several decentralised trial components, including electronic consent,^28^ sponsor oversight and monitoring^27^ and sponsor access to electronic health records.^33^ Collectively, these sources indicate that several decentralised activities are already permissible within the UK regulatory framework, provided that appropriate governance, oversight and data protection measures are in place.

Two guidance documents issued during the COVID-19 pandemic introduced temporary regulatory flexibilities to support trial continuity, including remote monitoring and direct-to-participant delivery of IMPs.^34,35^ Although developed as short-term measures, these documents demonstrate that decentralised trial activities could be implemented safely and feasibly.

Policy papers^2,3,36^ position decentralisation as key to the future of UK clinical trial delivery, emphasising the need for more flexible, participant-centred models. The Health Research Authority (HRA) position statement^37^ provides a formal definition for decentralised trial methods and highlights sponsor responsibilities for risk assessment, oversight and stakeholder engagement.

Definitions of a ‘trial site’ differed across regulatory sources. The *Medicines for Human Use (Clinical Trials) Regulations 2004*^20^ and ICH harmonised guideline integrated addendum to *ICH E6(R1): Guideline for Good Clinical Practice ICH E6(R2*)^25^ define trial sites as physical locations. In contrast, the HRA Integrated Research Application System defines a site as a legal entity responsible for participant care, regardless of where activities occur.^26^ This distinction is particularly important for decentralised trials, where activities may occur outside traditional healthcare settings, as it provides a framework for determining legal responsibility for oversight, governance and participant safety in this scenario.

Important gaps remain, including the absence of a single consolidated framework and limited clarity regarding consumer-grade digital devices (eg, smartphones or fitness trackers) that have not been formally validated as medical devices, raising additional data-quality and data-protection considerations.

The COVID-19 era guidance documents from the MHRA and HRA remain publicly accessible; however, it is not fully clear whether the flexibilities they introduced can still be applied in trials today. Although these measures were intended as temporary responses to the pandemic, they showed that decentralised trial approaches could work in practice. Their relevance now is therefore likely to be context-dependent, with newer regulatory initiatives expected to replace or formalise them over time.

### Survey of UKCRC CTUs

Survey findings are presented descriptively, with qualitative themes (online supplemental file 5) summarised alongside quantitative results

The survey was distributed to 49 UKCRC-registered CTUs. One CTU submitted separate responses for oncology and non-oncology because these departments operated independently. These were therefore treated as separate responses for the purposes of analysis, resulting in a total of 27 responses from a possible 50, a response rate of 54% (27/50). Respondents represented a range of roles, most commonly Operations Directors (n=11), Heads of Trial Management (n=6) and Quality Assurance Managers (n=6) with additional responses from Trialists (n=2), a Head Statistician (n=1) and a Deputy Director (n=1). Three CTUs reported conducting oncology studies alone, 10 conducted non-oncology studies alone, and 14 conducted both.

**Figure 2:**
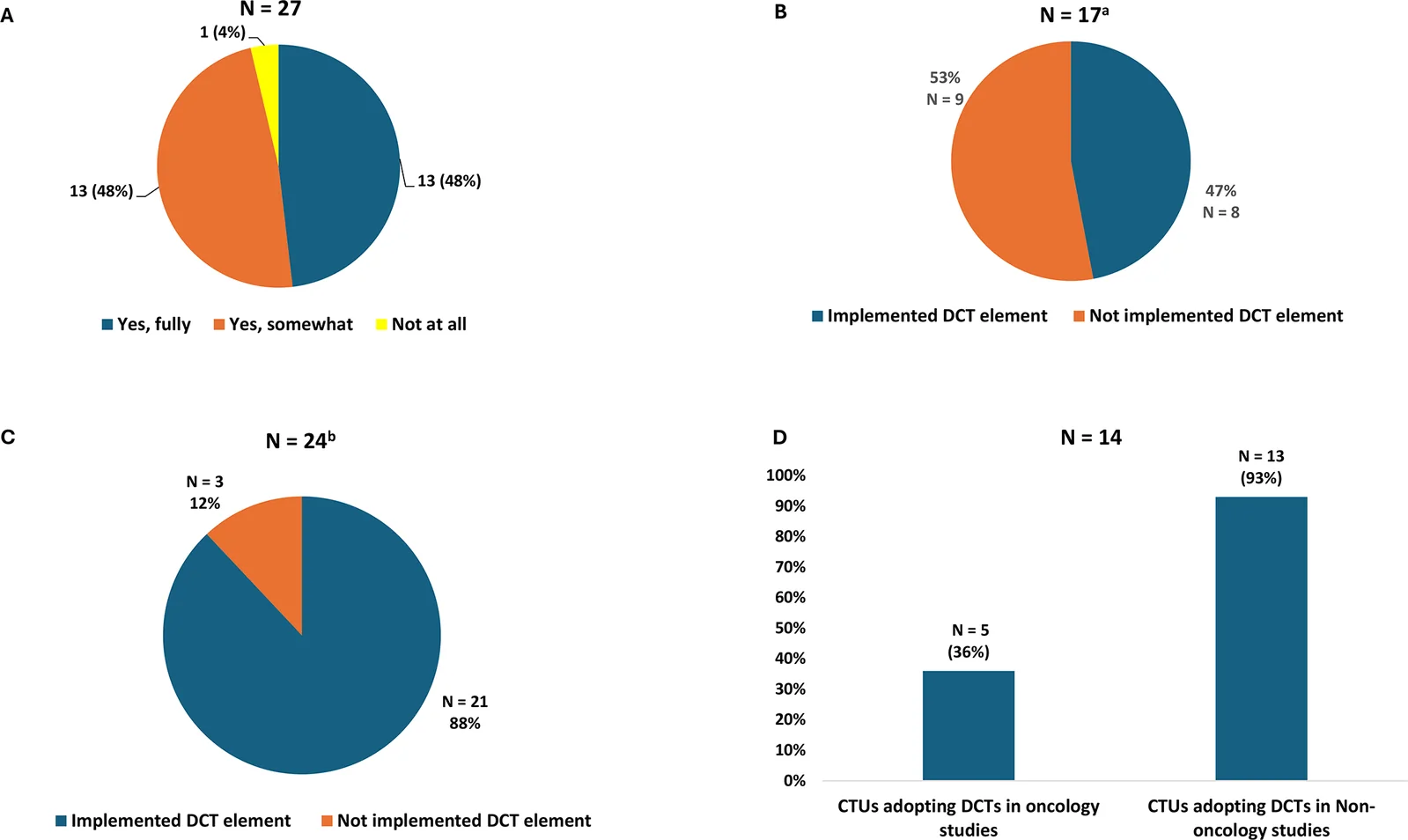
Awareness of decentralised trial methods among academic UK CTUs. (A) Familiarity of respondents with concept of DCTs; (B) DCT implementation among CTUs conducting interventional oncology studies, ^ᵃ^Three of these CTUs conduct oncology studies only; (C) DCT implementation among CTUs conducting interventional non-oncology studies, ^ᵇ^10 of these CTUs conduct non-oncology studies only; (D) comparison of DCT implementation among CTUs conducting both interventional oncology and non-oncology studies. CTUs, Clinical Trials Units; DCT, decentralised clinical trial.

Awareness of decentralised methods was high, with all but one respondent reporting at least some familiarity. Just under half of respondents, 48% (13/27) reported being fully familiar with the concept (figure 2A). When asked whether there should be consensus on terminology used to describe DCTs (eg, ‘remote’, ‘virtual’, ‘patient-centric’), 63% (17/27) of respondents agreed, while 33% (9/27) were neutral and one respondent did not provide an answer. Uptake of decentralised approaches varied markedly by study type. Among CTUs delivering interventional oncology studies, 47% (8/17) reported having implemented decentralised trial elements (figure 2B), compared with 88% (21/24) of CTUs delivering non-oncology studies (figure 2C). Among CTUs conducting both oncology and non-oncology studies, use of decentralised approaches was reported more frequently in non-oncology trials (figure 2D).

Decentralised approaches were applied across multiple trial processes, with follow-up data collection reported most frequently, particularly in non-oncology studies. Other areas included consent, participant screening, outcome assessments and intervention delivery. Most respondents reported no major concerns during regulatory or ethical approval processes. However, two CTUs described specific challenges, relating to eConsent regulatory compliance and to obtaining section 251 exemption for large-scale routine data access.

**Figure 3:**
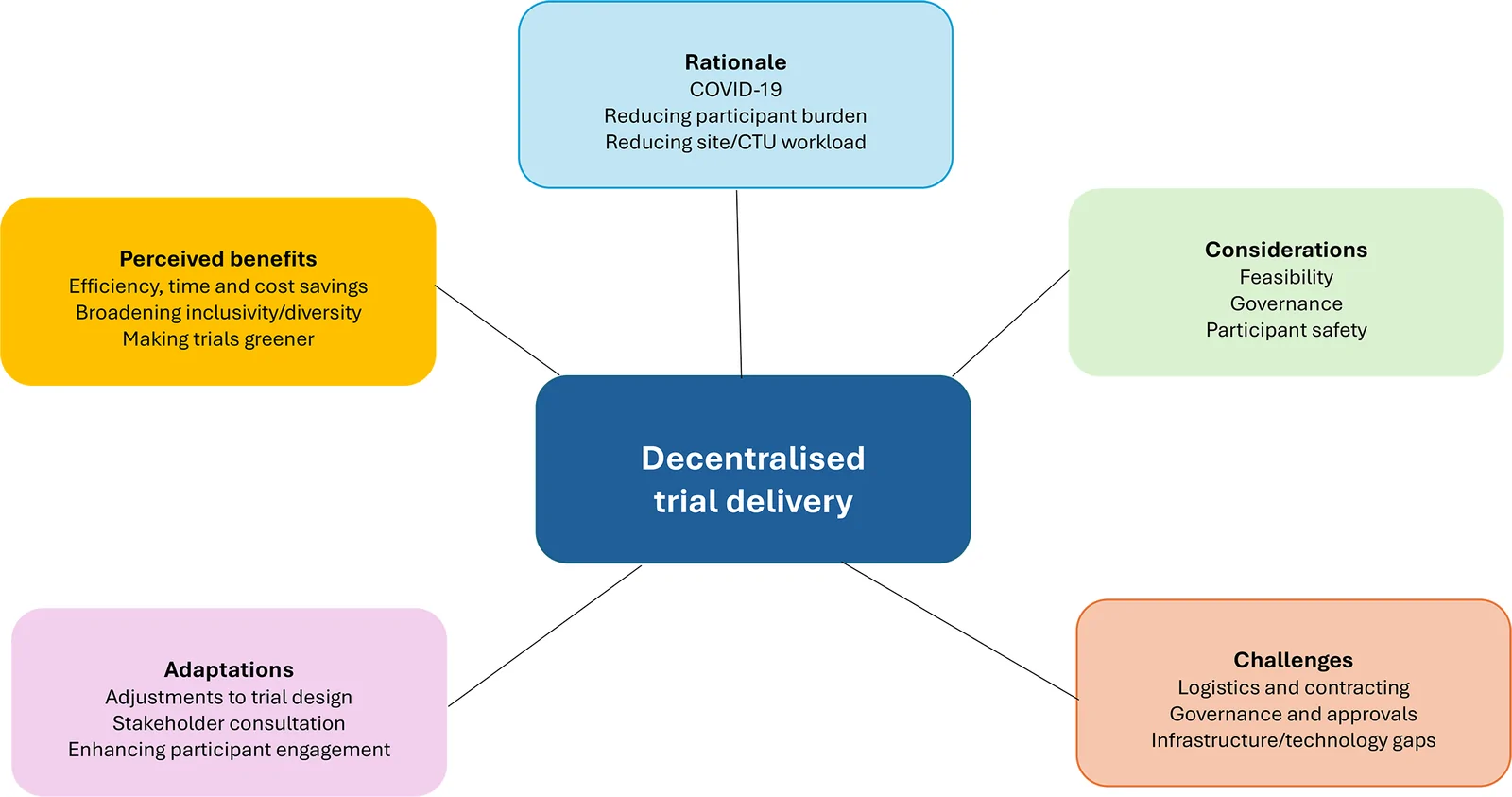
Thematic map of academic CTU perspectives on decentralised trials. CTU, Clinical Trials Unit.

Qualitative responses (figure 3; online supplemental file 5) indicated that the COVID-19 pandemic was a strong driver for DCT implementation, although some CTUs reported prior experience with digital approaches. Decentralisation was viewed as attractive due to its potential to reduce participant burden, increase flexibility and improve efficiency, particularly where remote follow-up and electronic data capture reduced administrative workload. However, these benefits were context-dependent, with some respondents noting that decentralised approaches could also be resource-intensive. Adoption was influenced by study-specific considerations, including feasibility, governance requirements and participant safety.

Respondents also described challenges including logistical and contracting complexities, data management and regulatory hurdles and limitations in infrastructure and technology. Participant-level barriers such as digital literacy and access were noted. Hybrid consent options, simplifying participant-facing materials and engaging regulators and stakeholders early were used to address these issues. However, some uncertainty remained around how to best define decentralisation, how to manage privacy and security in large-scale studies and whether funders would support any additional associated costs.

## Discussion

In this study, we examined decentralisation in UK oncology trials by integrating findings from an international literature review, regulatory and policy documents and a national survey of academic CTUs. Overall, the findings indicate growing interest in decentralised approaches but highlight challenges to their implementation in oncology, including a limited oncology-specific evidence base, fragmented regulatory guidance, operational challenges reported by CTUs relating to contracts, infrastructure, data management and staffing, and variation in how DCT models are defined and classified within the literature.

Our review suggests that decentralisation in oncology has mostly been evaluated in small studies focused on feasibility or assessment of process outcomes. A common limitation is the lack of randomised controlled comparisons evaluating decentralised versus conventional trial delivery. None of the included studies randomised participants between decentralised and traditional pathways. Only one study incorporated a contemporaneous comparator for a decentralised component; this was based on participant preference and did not incorporate adjustment for confounding in the analysis.^12^ Although several studies reported findings consistent with improved accessibility and efficiency, such as reduced travel burden,^12^ streamlined enrolment,^13^ better data completeness^13,14,16^ and high participant satisfaction,^15^ the absence of randomised or controlled comparisons means there is potential for bias and confounding. It is therefore difficult to be certain that these benefits were directly due to decentralisation.

Existing literature highlights variability in how decentralised trial models are defined and classified,^5^ and this lack of standardisation was also reflected in survey responses. While 63% supported consensus on DCT terminology, one-third were neutral, suggesting ongoing uncertainty about the scope and meaning of decentralisation. Some frameworks, including recent Food and Drug Administration (FDA) guidance, define DCTs broadly as studies incorporating decentralised elements but do not distinguish between fully decentralised and hybrid designs.^6^ Others conceptualise decentralisation along a continuum or explicitly distinguish hybrid models that combine remote and site-based activities.^38,39^

Definitions of ‘fully decentralised’ also vary; while some frameworks consider trials requiring any in-person visits as hybrid, others consider trials fully decentralised provided activities are conducted away from a central trial site, even if participants attend local facilities for procedures such as imaging or blood tests. Consequently, studies such as WISDOM and WJOG15221M/ALLBREAK may be classified differently depending on the framework applied. Although this variation is unlikely to affect implementation directly, it may influence interpretation of the evidence base by affecting which studies are considered decentralised, complicating comparisons across reviews. Describing specific decentralised elements may be more informative than applying binary labels such as ‘fully decentralised’ or ‘hybrid’.

The small number of studies included in the primary synthesis likely reflects several factors. First, our inclusion criteria deliberately focused on studies that formally evaluated decentralised methods as part of trial delivery, and such evaluations remain limited in oncology. Second, inconsistent terminology may mean that relevant decentralised elements are not always clearly described or indexed as such. Therefore, the limited number of eligible studies should not be interpreted as evidence of limited digital innovation in oncology, but rather as limited published evaluation of decentralised trial delivery within oncology.

The policy and legislative review suggests that, although relevant UK regulation exists, fragmentation across multiple sources may hinder implementation. Existing guidance supports several decentralised activities, including electronic consent, remote monitoring, sponsor access to electronic health records and COVID-19 era flexibilities demonstrated that more decentralised approaches could be implemented safely and feasibly. However, the framework is likely difficult to navigate in practice. Definitions of a ‘trial site’ differ across sources, and this is important for establishing accountability and appropriate oversight when trial activities occur outside traditional settings. While the revised UK Clinical Trials Regulations which came into effect on 28 April 2026 may help address some of this ambiguity, there remains no single consolidated framework for conducting decentralised trials in the UK.

In contrast to the USA, where the FDA published dedicated DCT guidance in 2024,^6^ and Europe, where the European Medicines Agency recommendation paper provides guidance on decentralised elements,^7^ as well as Denmark, which amended its Clinical Trials Regulations to permit direct-to-participant IMP delivery,^40^ the UK framework remains fragmented. Addressing these gaps could help reduce some of the barriers reported by CTUs in this study and bring policy ambitions closer to practice.

The CTU survey added a practice-based perspective. Awareness was high but use of decentralised approaches was lower in oncology than in non-oncology trials and was most often applied to participant follow-up data collection. CTUs described operational challenges including contracting, data management, infrastructure and staffing demands, as well as participant-level barriers such as digital access and literacy. These findings suggest that decentralisation may reduce burden for participants and sites while shifting workload to CTUs and central trial teams. Overall, CTUs appear motivated to adopt decentralised approaches but face both internal and external constraints. While some challenges, including staffing, data management and operational processes may be addressed at CTU level, regulatory uncertainty, infrastructure limitations and funding considerations require a systems-level approach.

These challenges resonate with UK policy commitments to strengthen infrastructure for decentralised trial delivery, including digital systems and model agreements to support innovative trial designs.^36^ While these initiatives directly target some of the challenges highlighted by CTUs, particularly around contracts and digital infrastructure, survey responses suggest progress has been slow and the impact on routine trial operations is not yet being felt. Many CTUs were adapting by offering hybrid consent options, simplifying participant-facing materials and engaging regulators and other stakeholders early.

For oncology, the challenges are distinct. Trials are often complex, involve high-risk interventions, require intensive safety monitoring and depend on protocol-specific imaging and laboratory assessments. Feasibility of decentralised approaches is likely to vary by trial phase and treatment modality. Early-phase studies may be less amenable to decentralisation because of dose escalation, pharmacokinetic or pharmacodynamic sampling and close toxicity monitoring. Later-phase and prevention studies may offer greater opportunities for remote visits, remote electronic consent, patient-reported outcomes, local blood tests and decentralised follow-up, particularly where interventions have established safety profiles.

Feasibility may also differ across systemic therapy, radiotherapy and surgery trials, as each has different requirements for specialist equipment, procedural expertise, IMP handling and safety oversight. These factors may explain the lower uptake in oncology CTUs. Participants in oncology trials also face a serious illness, and their views on decentralised approaches may differ from those in healthier or lower-risk populations. While there are qualitative studies exploring participant experiences in DCTs,^41,42^ to our knowledge there is no published patient and public involvement work exploring acceptability and preferences specifically among people with cancer.

This study has strengths in drawing on three complementary perspectives and focusing on oncology, where evidence is sparse. However, stakeholder perspectives were limited to the responding academic UK CTUs; patients, clinicians, regulators or industry representatives were not included. The findings therefore primarily reflect researcher and operational perspectives and may not fully capture patient priorities or concerns or be nationally representative of decentralised trial adoption across all UK CTUs. The survey instrument was not formally validated, as it was designed as a one-off descriptive assessment.

A further limitation is that only 27 of 49 CTUs responded to the survey, which may limit representativeness and introduces the possibility of non-response bias. To assess this, we reviewed publicly available profiles of non-responding CTUs. Among these, 12 (52%) of 23 listed cancer among their disease research areas, compared with 17 (63%) of 27 of responding CTUs. This suggests that CTUs with a cancer research focus were not markedly under-represented or over-represented among respondents, although non-response bias cannot be excluded.

The literature review was not prospectively registered and did not include formal quality or risk-of-bias assessment. This limits assessment of the reliability of individual study findings. However, given the limited and heterogeneous evidence base, which consisted primarily of non-comparative feasibility and implementation studies, a descriptive narrative synthesis was considered appropriate.

In addition, the UK policy and regulatory environment is evolving rapidly. As a result, the findings of the policy review represent a snapshot of the regulatory landscape at the time the review was conducted and may not fully reflect subsequent developments. The Saving and Improving Lives^3^ and its 2022–2025 implementation plan^36^ were launched under the previous Conservative government and although the core commitments are reflected in subsequent strategy updates,^43^ clear delivery timelines are not always explicitly stated. At the time of manuscript submission (16 April 2026), implementation of the revised UK Clinical Trials Regulations was imminent, and the regulations subsequently came into force on 28 April 2026. This includes replacing the term ‘trial site’ with ‘trial location’, reflecting a broader definition that allows trial activities to take place beyond traditional clinical settings, including in participants’ homes or mobile units, and may further support the implementation of decentralised approaches.^44^

In summary, the three strands highlight progress but persistent gaps between policy intent and practice. The international evidence base remains limited and patient perspectives in decentralised oncology trials are under-represented. Although aspects of our study focused on the UK, these findings are likely relevant to other jurisdictions seeking to implement decentralised approaches in oncology trials. Future work should explore multistakeholder perspectives, including patients, clinicians, regulators and trial delivery teams, to identify practical pathways for wider implementation of decentralised approaches in oncology trials.

## Supplementary material

10.1136/bmjonc-2026-001145 online supplemental file 1

10.1136/bmjonc-2026-001145 online supplemental file 2

10.1136/bmjonc-2026-001145 online supplemental file 3

10.1136/bmjonc-2026-001145 online supplemental file 4

10.1136/bmjonc-2026-001145 online supplemental file 5

10.1136/bmjonc-2026-001145 online supplemental file 6

## Data Availability

Data are available upon reasonable request.
