# Supplementary material for: Decentralised Oncology Trials in the UK: Evidence, Regulation and Academic Clinical Trials Units’ Perspectives
Source: BMJ Oncol. 2026 Aug 20;5(2):e001145. doi: 10.1136/bmjonc-2026-001145 (PMC13504880; doi:10.1136/bmjonc-2026-001145)
Supplement: Supplementary file 1 [file bmjonc-5-2-s001.pdf]

Supplementary File 1. Full Ovid search strategy

1. decentralised trial*.tw.
2. decentralized trial*.tw.
3. remote.tw.
4. remote trial*.tw.
5. virtual trial*.tw.
6. digital trial*.tw.
7. patient-centric trial*.tw.
8. hybrid trial*.tw.
9. site-less trial*.tw.
10. direct-to-patient.tw.
11. direct-to-participant.tw.
12. direct-to-subject.tw.
13. patient-centric trial.tw.
14. patient-centric study.tw.
15. patient centered trial.tw.
16. patient centered study.tw.
17. patient centred trial.tw.
18. patient centred study.tw.
19. home-based trial.tw.
20. digital medicine.tw.
21. e-medicine.tw.
22. emedicine.tw.

23.	online trial.tw.
24.	internet based trial.tw.
25.	web-based trial.tw.
26.	tele-visit.tw.
27.	e-health.tw.
28.	ehealth.tw.
29.	electronic health.tw.
30.	m-health.tw.
31.	mobile health.tw.
32.	mobile application*.tw.
33.	wearable electronic device*.tw.
34.	e-consent.tw.
35.	econsent.tw.
36.	electronic consent.tw.
37.	1 or 2 or 4 or 5 or 6 or 7 or 8 or 9 or 10 or 11 or 12 or 13 or 14 or 15 or 16 or 17 or 18 or 19 or 20 or 21 or 22 or 23 or 24 or 25 or 26 or 27 or 28 or 29 or 30 or 31 or 32 or 33 or 34 or 35 or 36
38.	cancer*.tw.
39.	malignanc*.tw.
40.	tumo?r*.tw.
41.	neoplasm*.tw.
42.	38 or 39 or 40 or 41
43.	clinical trial*.tw.
44.	research stud*.tw.

45.	interventional clinical trial*.tw.
46.	interventional research stud*.tw.
47.	exp Clinical Trials as Topic/
48.	43 or 44 or 45 or 46 or 47
49.	37 and 42 and 48
