# Supplementary material for: Decentralised Oncology Trials in the UK: Evidence, Regulation and Academic Clinical Trials Units’ Perspectives
Source: BMJ Oncol. 2026 Aug 20;5(2):e001145. doi: 10.1136/bmjonc-2026-001145 (PMC13504880; doi:10.1136/bmjonc-2026-001145)
Supplement: Supplementary file 2 [file bmjonc-5-2-s002.pdf]

Supplementary File 2. Literature review data extraction fields

**Citation and identification**	Full title of article
	Authors
	Year of publication
	Study full title
	Study short title
	Trial identifier (if applicable)
	Sponsor
**Study summary**	Brief study description
	Start date
	End date (est. or actual)
	Location
	Study status
**Study design and population**	Study design
	Patient population
	Disease setting
	Main clinical intervention
	Primary endpoint
	No. of screened/consented/randomised participants
**DCT characteristics**	DCT design
	What was the DCT component
	Rationale for decentralisation/for evaluating the digital innovation
	How was it decentralised?
**Evaluation of the DCT approach**	Outcomes used to evaluate DCT approach
	Details of evaluation
	Results of evaluation
**Implementation**	Facilitators
	Benefits described
	Challenges described
	Equity/access considerations (if indicated)
