# Supplementary material for: Decentralised Oncology Trials in the UK: Evidence, Regulation and Academic Clinical Trials Units’ Perspectives
Source: BMJ Oncol. 2026 Aug 20;5(2):e001145. doi: 10.1136/bmjonc-2026-001145 (PMC13504880; doi:10.1136/bmjonc-2026-001145)
Supplement: Supplementary file 3 [file bmjonc-5-2-s003.pdf]

Supplementary File **3.** Legislation review extraction fields

Decentralisable Domains	Specific activity	Issuing Authority	Opportunities	Barriers	Gaps
**Trial set-up**	Site				
	SIV				
**Recruitment**	Consent				
	Screening				
**Intervention delivery & Follow-up**	IMP administration				
	DtP IMP shipment				
	Safety monitoring				
	On-treatment outcome assessments				
	Long-term follow-up				
**Data processing & handling**	Data capture				
	rSDV				
	Data flow/privacy				
**Operations**	Oversight				
	Monitoring				

Abbreviations: SIV, Site initiation visit, IMP, Investigational medicinal product, DtP, direct-to-participant, rSDV, remote source data verification
