# Supplementary material for: Decentralised Oncology Trials in the UK: Evidence, Regulation and Academic Clinical Trials Units’ Perspectives
Source: BMJ Oncol. 2026 Aug 20;5(2):e001145. doi: 10.1136/bmjonc-2026-001145 (PMC13504880; doi:10.1136/bmjonc-2026-001145)
Supplement: Supplementary file 4 [file bmjonc-5-2-s004.pdf]

The Institute of Cancer Research

Supplementary File 4. Survey Exploring Decentralisation of Cancer Clinical Trials in the UK

Traditionally, many clinical trials typically involve a physical study site at which all trial related activity takes place and to which participants need to physically travel to. Decentralised clinical trials (DCTs) are trials that aim to move away from this approach into one where the need for participants to visit trial sites is reduced or completely eliminated, through local delivery of trial components. This might be at the participant's home, at a local healthcare setting, or via remote/electronic means. In fully DCTs, all elements of trial activity are conducted externally to the primary trial site. However, it is recognised that decentralisation is likely a spectrum and, for many trials, a hybrid model may be most appropriate.

DCTs are sometimes alternatively referred to as 'remote', 'digital', 'virtual' or 'patient-centric' trials and may incorporate the use of innovative digital tools and technologies such as e-consent, telemedicine or use of wearable devices, for example, to carry out some or all aspects of participant recruitment, treatment, outcome assessment and follow-up.

The use of DCTs has grown organically as well as in response to the COVID-19 pandemic, where the need for reduced physical contact motivated the adoption of remote methods. However, whilst DCTs offer several potential benefits, there are also challenges to be addressed. To our knowledge, these considerations are yet to be fully evaluated within oncology clinical trials.

The primary aim of this survey is therefore to evaluate the extent to which decentralisation has been integrated into interventional oncology clinical trials to date and to evaluate the practicalities, perceived benefits, and challenges encountered. A further aim is to assess understanding of DCTs within UKCRC CTUs.

General Background Questions

Q1. Name of person completing this survey: *

Q2. Role of person completing this survey: *

Q3. Please select CTU where you work:

- ◯ Barts and The London Pragmatic CTU
- ◯ Barts Clinical Trials Unit
- ◯ Birmingham CTU
- ◯ Brighton & Sussex Clinical Trials Unit
- ◯ Bristol Trials Centre
- ◯ CaCTUS
- ◯ Cambridge Clinical Trials Unit
- ◯ Cambridge Epidemiology & Trials Unit
- ◯ CRUK & UCL Cancer Trials Centre
- ◯ Cancer Research UK Clinical Trials Unit (CRCTU)
- ◯ Centre for Healthcare Randomised Trials (CHaRT)
- ◯ Centre for Trials Research
- ◯ Comprehensive Clinical Trials Unit at UCL
- ◯ Derby Clinical Trials Support Unit
- ◯ Diabetes Trials Unit Oxford
- ◯ Edinburgh Clinical Trials Unit
- ◯ Exeter Clinical Trials Unit
- ◯ Glasgow CTU
- ◯ Imperial Clinical Trials Unit
- ◯ Intensive Care National Audit & Research Centre ICNARC
- ◯ Keele CTU
- ◯ King’s Clinical Trials Unit at King’s Health Partners
- ◯ Lancashire Clinical Trials Unit
- ◯ Leeds CTRU
- ◯ Leicester Clinical Trials Unit
- ◯ Liverpool Clinical Trials Centre
- ◯ London School of Hygiene & Tropical Medicine CTU
- ◯ MRC CTU at UCL
- ◯ Newcastle CTU
- ◯ NHS Blood and Transplant Clinical Trials Unit
- ◯ North Wales Organisation for Randomised Trials in Health (& Social Care) – NWORTH

- Northern Ireland CTU
- Norwich CTU
- Nottingham CTU
- NPEU CTU
- Clinical Trial Service Unit and Epidemiological Studies Unit
- OCTRU – Oxford Clinical Trials Research Unit
- Oxford Primary Care & Vaccines Collaborative CTU
- Papworth Trials Unit Collaboration
- Peninsula CTU
- PRIMENT CTU at UCL
- Royal Marsden Clinical Trials Unit
- Sheffield Clinical Trials Research Unit
- Southampton CTU
- Swansea Trials Unit
- Tayside CTU
- The ICR-CTSU
- Warwick CTU
- York Trials Unit
- Other

***Q3a*. If selected 'Other' in Q3, please specify:**

Q4. Which disease areas does your CTU conduct studies into: *

Please select all that apply.

- Antimicrobial Resistance
- Artificial Intelligence
- Blood (excluding cancer)
- Cancer
- Cardiovascular
- Dementias and Neurodegenerative Disease

- [ ] Diabetes
- [ ] Eye (excluding cancer)
- [ ] Ear
- [ ] Generic Health Relevance
- [ ] Infection
- [ ] Inflammation and Immune system
- [ ] Mental Health
- [ ] Metabolic & Endocrine
- [ ] Musculoskeletal
- [ ] Neurology
- [ ] Oral & Gastrointestinal (excluding cancer)
- [ ] Primary Care
- [ ] Renal & Urogenital (excluding cancer)
- [ ] Reproductive & Childbirth
- [ ] Respiratory (excluding cancer)
- [ ] Skin (excluding cancer)
- [ ] Stroke
- [ ] Other

Q4a. You've selected 'Cancer' in Q4 above, please specify type: *

Please select all that apply.

- [ ] Malignant haematology
- [ ] Solid organ tumours

Q4b. You've selected 'Other' in Q4, please specify:

Q5. Study phases conducted at your CTU:

Please select all that apply.

- [ ] Phase I
- [ ] Phase II

- [ ] Phase III
- [ ] Phase IV

Q6. Study types conducted:

Please select all that apply.

- [ ] CTIMP
- [ ] Non-CTIMP

Q6a. For non-CTIMPs, please specify study types:

Please select all that apply.

- [ ] Surgical
- [ ] Radiotherapy
- [ ] Medical device
- [ ] Psychosocial
- [ ] Other

Q6b. You've selected 'Other' in *Q6a*, please give detail here:

Understanding what Decentralised Clinical Trials (DCTs) mean to CTUs

For the purposes of this and subsequent sections of this survey, please think of decentralisation as described within the introductory text.

Q7. Are you familiar with the concept of decentralisation within clinical trials?

- ◯ Yes, fully
- ◯ Yes, somewhat
- ◯ Not at all

Q8. There are currently different nomenclatures for DCTs such as 'remote', 'virtual' or 'patient-centric' trials. In your opinion, should there be consensus on a single terminology when referring to DCTs?

- ◯ Yes
- ◯ No
- ◯ Neutral

Q9. How important are the below when considering decentralisation within interventional clinical trials:

Please note that dependent on screen size, you may need to scroll horizontally using the bar beneath the grid to view all the available options.

Risk categorisation of the intervention and extent of risk-mitigating strategies

- ☐ Not at all important
- ☐ Slightly important
- ☐ Important
- ☐ Fairly important
- ☐ Very important
- ☐ No opinion

Type and nature of the intervention (e.g. IMP or non-IMP, pharmaceutical form, method of administration)

- ☐ Not at all important
- ☐ Slightly important
- ☐ Important
- ☐ Fairly important
- ☐ Very important
- ☐ No opinion

Disease type (e.g. chronic conditions)

- [ ] Not at all important
- [ ] Slightly important
- [ ] Important
- [ ] Fairly important
- [ ] Very important
- [ ] No opinion

Nature and extent of patient safety monitoring required

- [ ] Not at all important
- [ ] Slightly important
- [ ] Important
- [ ] Fairly important
- [ ] Very important
- [ ] No opinion

Complexity of the trial protocol

- [ ] Not at all important
- [ ] Slightly important
- [ ] Important
- [ ] Fairly important
- [ ] Very important
- [ ] No opinion

Operational considerations (e.g. oversight, logistics)

- [ ] Not at all important
- [ ] Slightly important
- [ ] Important
- [ ] Fairly important
- [ ] Very important
- [ ] No opinion

Availability of technologies that have been validated and comply with regulatory standards

- [ ] Not at all important
- [ ] Slightly important
- [ ] Important
- [ ] Fairly important
- [ ] Very important
- [ ] No opinion

Extent of data collection requirements (i.e. number and type of endpoints and timepoints)

- [ ] Not at all important
- [ ] Slightly important
- [ ] Important
- [ ] Fairly important
- [ ] Very important
- [ ] No opinion

Participant preference/acceptability

- [ ] Not at all important
- [ ] Slightly important
- [ ] Important
- [ ] Fairly important
- [ ] Very important
- [ ] No opinion

Participant convenience/burden (e.g. travel costs, travel distance)

- [ ] Not at all important
- [ ] Slightly important
- [ ] Important
- [ ] Fairly important
- [ ] Very important
- [ ] No opinion

Site burden/resource-use

- [ ] Not at all important
- [ ] Slightly important
- [ ] Important
- [ ] Fairly important
- [ ] Very important
- [ ] No opinion

CTU burden/resource-use

- [ ] Not at all important
- [ ] Slightly important
- [ ] Important
- [ ] Fairly important
- [ ] Very important
- [ ] No opinion

Other (please elaborate below)

- [ ] Not at all important
- [ ] Slightly important
- [ ] Important
- [ ] Fairly important
- [ ] Very important
- [ ] No opinion

Q9a. If selected 'Other' in Q9 above, please provide more detail:

Please skip and move on to next question if this does not apply.

Existing decentralised approaches within interventional clinical trials (non-oncology)

This section of the survey further explores aspects that have been decentralised within non-oncology trials. Please note that for CTUs conducting only oncology studies, due to functional limitations within the survey builder, this section will still be displayed. Please choose the 'N/A' option in Q10 and move to the next section.

Q10. Which aspects below have been decentralised within your existing interventional non-oncology trials?

For CTUs conducting both oncology and non-oncology studies, please answer pertaining to non-oncology trials only in this section.

- ☐ Trial Set-up
- ☐ Participant identification/Screening
- ☐ Consent
- ☐ Intervention delivery
- ☐ On-treatment outcome assessments
- ☐ Follow-up data collection
- ☐ Other
- ☐ None
- ☐ N/A (my CTU only conducts oncology trials or I only work on oncology trials)

Q10a. Please detail here what specific aspects of **'Trial set-up'** have been decentralised, what the key factors contributing to your decision to decentralise this were, key practical considerations in implementing this approach, benefits realised and any challenges faced.

Q10b. Regarding **'Participant identification/Screening'**, what aspects have been decentralised, what key factors contributed to your decision to decentralise this, what were the key practical considerations in implementing this approach, benefits realised and any challenges faced.

Q10c. Please detail here what key factors contributed to your decision to implement **remote consent**, what the key practical considerations were, benefits realised and any challenges faced.

Q10d. Please detail here what key factors contributed to your decision to decentralise **'Intervention delivery',** what the key practical considerations were, benefits realised and any challenges faced.

Q10e. Please detail here what key factors contributed to your decision to decentralise **'On-treatment outcome assessments',** what the key practical considerations were, benefits realised and any challenges faced.

Q10f. Please detail here what key factors contributed to your decision to decentralise **'Follow-up data collection'**, what the key practical considerations were, benefits realised and any challenges faced.

Q10g. If selected **'Other'**, please detail here what was decentralised, what key factors contributed to your decision to decentralise it, what the key practical considerations were, benefits realised and any challenges faced.

Existing decentralised approaches within interventional clinical trials (oncology)

This section of the survey further explores aspects that have been decentralised specifically within oncology trials and will only be displayed if you indicated your CTU conducts studies into solid organ tumours.

Q11. Which aspects below have been decentralised within your existing interventional oncology trials?

- ☐ Trial Set-up
- ☐ Participant identification/Screening
- ☐ Consent
- ☐ Intervention delivery
- ☐ On-treatment outcome assessments
- ☐ Follow-up data collection
- ☐ Other
- ☐ None

Q11a. Please detail here what specific aspects of **'Trial set-up'** have been decentralised, what key factors contributed to your decision to decentralise this, what were the key practical considerations in implementing this approach, benefits realised and any challenges faced.

Q11b. Regarding **'Participant identification/Screening'**, what aspects have been decentralised, what key factors contributed to your decision to decentralise this, what were the key practical considerations in implementing this approach, benefits realised and any challenges faced.

Q11c. Please detail here what key factors contributed to your decision to implement **remote consent**, what the key practical considerations were, benefits realised and any challenges faced.

Q11d. Please detail here what key factors contributed to your decision to decentralise **'Intervention delivery',** what the key practical considerations were, benefits realised and any challenges faced.

Q11e. Please detail here what key factors contributed to your decision to decentralise **'On-treatment outcome assessments',** what the key practical considerations were, benefits realised and any challenges faced.

Q11f. Please detail here what key factors contributed to your decision to decentralise **'Follow-up data collection'**, what the key practical considerations were, benefits realised and any challenges faced.

Q11g. If selected **'Other'**, please detail here what was decentralised, what key factors contributed to your decision to decentralise it, what the key practical considerations were, benefits realised and any challenges faced.

Regulatory & Ethical Approvals

Q11. Did any aspects of decentralisation cause concerns at regulatory and/or ethical approval stage?

If your CTU has not adopted any decentralised approaches, please select 'not applicable' here.

- ○ Yes
- ○ No
- ○ Not applicable

Q11a. Please detail here what the concerns were:

Q11b. How did you overcome these concerns?

Looking ahead

Q12. In the planned revision of the UK legislation/regulatory framework, the MHRA are proposing to introduce a notification scheme for low-intervention trials, whereby only ethical approval would be required to initiate such a study. Do you think this proposed change could facilitate the embracing of innovative approaches like decentralisation?

- ◯ Yes
- ◯ No
- ◯ Unsure

Q13. We would like to acknowledge your participation in this survey by including you in publication outputs arising from it. We may also wish to contact you for further discussions. If you agree to be acknowledged and contacted, please provide a preferred contact email address:

Q14. Any additional comments?
