# Supplementary material for: Decentralised Oncology Trials in the UK: Evidence, Regulation and Academic Clinical Trials Units’ Perspectives
Source: BMJ Oncol. 2026 Aug 20;5(2):e001145. doi: 10.1136/bmjonc-2026-001145 (PMC13504880; doi:10.1136/bmjonc-2026-001145)
Supplement: Supplementary file 5 [file bmjonc-5-2-s005.pdf]

Supplementary File 5. Thematic analysis of CTU survey free-text responses

Name	Description	Files	References
**Theme 1. Rationale for adopting a decentralised approach**	**This theme represents the underlying motives provided by respondents for adopting a decentralised trial approach.**	**15**	**28**
Sub-theme 1. COVID-19 as a catalyst	The pandemic was a key driver of adoption	6	8
Sub-theme 2. Pre-existing practices and experience	Prior use of eConsent, online questionnaires, or other elements eased transition.	2	3
Sub-theme 3. Intervention characteristics	Decentralisation was felt to be well-suited to low-risk, self-managed interventions (e.g. eczema trial).	5	7
Sub-theme 4. Reducing participant burden	A strong rationale given was the potential to reduce participant burden.	6	6
Sub-theme 5. Reducing site and CTU workload	Some respondents saw decentralisation as a way to ease pressure on research sites and CTUs.	4	4
**Theme 2. Multifaceted considerations for adopting a decentralised approach**	**This theme reflects some of the factors CTUs considered when deciding whether, and how, to decentralise.**	**7**	**28**
Sub-theme 1. Feasibility and practical implementation	Respondents noted that feasibility was study-specific, depending on the data required and available systems. A recurring issue was clarity around the division of responsibilities between CIs, PIs, sponsors, and usual care teams. Existing institutional infrastructure, such as licensed survey software, was also cited as supporting practical delivery.	4	6

Name	Description	Files	References
Sub-theme 2. Regulatory compliance and governance requirements	Respondents highlighted multiple governance considerations, for example, ensuring eConsent adhered to MHRA/HRA requirements and that participant identity could be verified. Other considerations included insurance and indemnity and management of source data.	3	10
Sub-theme 3. Participant safety and risk management	Respondents considered participant safety central to decisions about decentralisation. Low-risk studies were viewed as appropriate for remote screening, consent, and initiation, whereas higher-risk studies were not.	6	10
**Theme 3. Perceived benefits of decentralised trial approaches**	**This theme represents the benefits of DCTs reported by respondents**	**11**	**30**
Sub-theme 1. Benefits for Sites and CTUs	Respondents perceived that decentralisation could ease site and CTU workload	3	3
Sub-theme 2. Efficiency, time and cost savings	Decentralised approaches were perceived as more efficient and cost-saving, with respondents citing reduced travel and fewer expenses for site set-up.	9	12
Sub-theme 3. Broadening inclusivity and diversity	Respondents highlighted that decentralisation could allow access to wider and more diverse populations, for example through NHS Digital recruitment or social media.	5	5
Sub-theme 4. Making trials 'greener'	Some respondents anticipated environmental benefits, suggesting that reducing participant travel could lower the carbon footprint of trials.	2	2

Name	Description	Files	References
Sub-theme 5. Improving participant experience	Decentralisation was thought to improve participant experience by making participation more convenient and flexible, reducing the need for travel, and offering more choice in how trial activities were completed.	6	8
**Theme 4. Challenges in implementing decentralised trial approaches**	**This theme represents the challenges of DCT implementation reported by respondents**	**7**	**27**
Sub-theme 1. Practical and logistical challenges	Respondents noted difficulties with contracting, documentation, and information sharing between sites, vendors, and coordinating teams. Additional challenges included managing procurement, shipping drugs to participants, and GP-related processes such as issuing letters or confirming participant details.	3	4
Sub-theme 2. Challenges with data management and regulatory compliance	Challenges included the cost, timeliness, and quality of real-world data; complexities of data access applications (e.g. s251, DARS/IGARD); and defining data responsibilities. Respondents also highlighted difficulties with archiving across dispersed teams, reliance on secure IT connections between organisations, and the lack of affordable compliant systems for remote consent.	3	6
Sub-theme 3. Limitations in infrastructure and technology	Respondents reported difficulties finding reliable e-tools for patient-reported outcomes and ensuring technical support. Problems arose from variable IT capacity across GP practices, system incompatibilities, and lack of affordable platforms meeting regulatory standards.	3	6
Sub-theme 4. Barriers to participant engagement and participation	Digital literacy, internet access, and language barriers were seen as obstacles to participation. Some participants found IVRT lines confusing,	3	5

Name	Description	Files	References
	and follow-up questionnaires were hard to complete remotely, leading to non-response and loss to follow-up.		
Sub-theme 5. Staffing constraints and capacity limitations	Respondents described decentralised approaches as resource heavy, requiring significant staff time for set-up, monitoring, and running searches. Difficulties included limited availability of trained site staff, challenges in building rapport when trial managers were more "hands off," and shortages of CTU staff with relevant experience.	3	6
**Theme 5. Adaptations and solutions**	**This theme represents a range of adaptations reported by respondents to support decentralised trial conduct.**	**5**	**16**
Sub-theme 1. Adjustments to trial design and delivery	Examples included removing the concept of traditional sites and investigators in a "citizen science" trial, couriering trial drugs to participants to avoid hospital visits, prescribing at home where supported by infrastructure, and integrating real-world data alongside CRF data.	2	4
Sub-theme 2. Consulting with stakeholders	Respondents highlighted proactive engagement with regulators, sponsors, NHS England, and quality assurance groups to agree on approaches and ensure compliance. Early dialogue was seen as key to resolving potential issues.	3	3
Sub-theme 3. Strategies to enhance participant engagement	Adaptations included offering multiple consent options (e.g. phone, paper, website), simplifying patient-facing materials, and developing clear websites and helplines to support understanding. Involving PPIE and behavioural scientists, and developing apps with user input, were also strategies to address participation barriers.	2	4

Name	Description	Files	References
**Theme 6. Ambiguities and uncertainties in decentralised trial implementation**	**Respondents highlighted uncertainties about what counts as decentralised, how to manage privacy and regulatory requirements in large-scale studies, and whether funders will support additional costs.**	**4**	**5**
