# Supplementary material for: Decentralised Oncology Trials in the UK: Evidence, Regulation and Academic Clinical Trials Units’ Perspectives
Source: BMJ Oncol. 2026 Aug 20;5(2):e001145. doi: 10.1136/bmjonc-2026-001145 (PMC13504880; doi:10.1136/bmjonc-2026-001145)
Supplement: Supplementary file 6 [file bmjonc-5-2-s006.pdf]

Supplementary File 6. Acknowledgement of Survey Respondents

The following survey respondents indicated their willingness to be acknowledged for their participation in this survey:

**Respondent**	**CTU Affiliation**
Joy Wiles	NPEU CTU
Joyce Thomson	Edinburgh Clinical Trials Unit
Andrea Harkin	CaCTUS
Puvan Nathan	York Trials Unit
Claire Daffern	Warwick CTU
Sarah Fielding	Papworth Trials Unit Collaboration
Lewis Beer	Tayside CTU
Gail Holland	Swansea Trials Unit
Matthew Burns	Lancashire Clinical Trials Unit
Elizabeth Denis	NWORTH
Jacqueline Nuttall	Southampton CTU
Jane Daniels	Nottingham CTU
Claire Snowdon	The ICR-CTSU
Vicki Barber	OCTRU
Michelle Sleeth	Barts Cancer Prevention Trials Unit
Laura White	Barts CTU (non-cancer)
Tahera Hussain	Barts and The London Pragmatic CTU
Catey Bunce	Royal Marsden Clinical Trials Unit
Angela Meade	MRC CTU at UCL
Emily Swinburne	Newcastle CTU
Felicia Ikeji	Comprehensive Clinical trials Unit at UCL (CCTU)
Jodi Taylor	Bristol Trials Centre
William Cragg	Leeds CTRU
Chris Rollinson	Peninsula CTU
